# Supplementary material for: Scalable, fast and accurate differential gene expression testing from millions of cells of multiple patients
Source: Nat Commun. 2026 Jun 19;17:7734. doi: 10.1038/s41467-026-74451-9 (PMC13434233; doi:10.1038/s41467-026-74451-9)
Supplement: Supplementary file 2 [file 41467_2026_74451_MOESM2_ESM.pdf]

nature portfolio

Peer Review File

Scalable, fast and accurate differential gene expression testing from millions of cells of multiple patients

Corresponding Author: Professor Giulio Caravagna

This file contains all reviewer reports in order by version, followed by all author rebuttals in order by version.

Version 0:

Reviewer comments:

Reviewer #1

(Remarks to the Author)
The authors present devil, a novel Bayesian framework for single-cell differential expression (scDE) testing that is both statistically rigorous and computationally scalable, capable of handling datasets with over 10 million cells across multiple patients. The method addresses critical limitations in existing approaches: conventional methods incorrectly assume cell-level independence, while pseudobulk method ignores cell-to-cell variation. The proposed method uses GPU-based variational inference for scalable estimation of regression coefficients. It also uses clustered sandwich covariance approach to address dependence of cells within patients, thereby avoids explosion of subject-specific parameters as seen in the mixed effect model. Overall, the authors provide a timely and important contribution to the field of scalable and accurate scDE analysis.

Comments for the authors:

1. It would be interesting to include comparisons with commonly used scDE tools such as FindMarkers (Seurat, Wilcoxon test) and MAST where feasible within a reasonable runtime threshold. This would give users practical intuition about when devil should be preferred over these widely adopted methods.

2. In cell-wise design (e.g., cell type marker identification), treating batch effects is essential in atlas-scale scDE. How does devil account for batch effects? A dedicated benchmark, either through simulations or real scRNA-seq datasets with known batch structure, would clarify devil’s robustness in these scenarios.

3. For broader adoption, the authors could provide additional test examples illustrating different experimental designs and application settings (with and without GPU acceleration). A more detailed user manual or tutorials on the GitHub repository would also be highly valuable.

4. In Fig. 2E (third panel), the MCC of glmGamPoi is strikingly low. Is this entirely attributable to its failure in false-positive control, or might there be additional factors contributing to this result? A deeper discussion would help readers interpret this discrepancy.

(Remarks on code availability)

Reviewer #2

(Remarks to the Author)
The authors present devil, a framework for scDE testing that accounts for statistical dependence between individual samples. They demonstrate that their method performs on par or better with current state-of-the-art approaches and offers a significant benefit in terms of computational performance. While the manuscript is of relevance and matches the scope of Nat Commun, some changes in the manuscript are necessary and major concerns are related to the code.
p. 4, l. 62: the notion of a design matrix X suggests that the considered covariates may only represent discrete levels (e.g., experimental conditions). Please clarify whether the approach can be extended towards continuous covariates (e.g., concentrations, age, …) and state this (possible) limitation in the discussion.

p. 4 l. 98: CPU and GPU vendor and model should be named in the main text, or at least include a reference to the supplementary table. Compiler version, CUDA version, R version etc. should be detailed in the appendix / supplements for reproducibility
p.4 l. 102: Does the high memory usage of glmGamPoi arise from the concept / methodology, or from a sub-optimal implementation? In case of the latter, this should be mentioned in the text for fair comparison.
p. 4 l. 103: The extrapolation method should be explained precisely. Is it a linear extrapolation, nonlinear etc.?
p. 7 l. 107: Please provide confirmation that devil yields the exact same p-values for the CPU and GPU implementation.
p. 7, l. 112: Why did the authors choose not to optimize the MLE inference before submission of the manuscript? Please elaborate in the manuscript how such optimization would be implemented.
p. 7, l. 135-144: Do the reported standard deviations refer to the simulation design reported in ll. 506? If so, please include a reference in the text
p. 12, l. 238: typo implementedd
p. 21, l. 611: Please perform a strong scaling experiment to demonstrate the scalability of devil and include it in the supplementary information. Based on the results in Suppl. Fig. 1B it would be expected that the strong scaling efficiency is limited by the CPU-part of the code.
Figure 3E: Why does the accuracy not improve further when adding more than ~100 markers?
Extended Figure 2A: Please add annotations for the cell type to the legend.

Comments on Code
• The Github repository does not contain any unit tests for any of the branches (CPU version, GPU version, Python bindings). It is strictly necessary to add tests where possible before acceptance of the manuscript.
• The GPU implementation in the devel branch appears to be incomplete. For example, the function “beta_fit_gpu_external” is used in beta.cpp but is not defined in the repository. Did I miss something? How can I inspect the code for the GPU implementation? Since the GPU version is central to the manuscript, it must be available and installable via GitHub.
• As a minor comment, the README.md should be updated for the Python branch (if it is considered stable, which I assume since it is mentioned on the main branch)

(Remarks on code availability)
Comments on Code
• The Github repository does not contain any unit tests for any of the branches (CPU version, GPU version, Python bindings). It is strictly necessary to add tests where possible before acceptance of the manuscript.
• The GPU implementation in the devel branch appears to be incomplete. For example, the function “beta_fit_gpu_external” is used in beta.cpp but is not defined in the repository. Did I miss something? How can I inspect the code for the GPU implementation? Since the GPU version is central to the manuscript, it must be available and installable via GitHub.
• As a minor comment, the README.md should be updated for the Python branch (if it is considered stable, which I assume since it is mentioned on the main branch)

Reviewer #3

(Remarks to the Author)
The authors present a novel approach for the differential expression analysis of single-cell RNA-seq data that takes into account the hierarchical design of multi-sample datasets and that is scalable to millions of cells. The approach is grounded in sound statistical approaches (sandwich estimator, mean-field variational inference) and is rendered attractive by the GPU implementation that makes it ultra scalable. The implementation in R allows users familiar with typical differential expression packages, such as edgeR and DESeq2, to easily adapt their pipeline. Overall, the proposed method seems a good addition to the tools available for differential expression in large, multi-sample datasets.

However, I have several major points to be addressed by the authors.

1. The authors refer to a design in which cells are collected from multiple patients per condition (in which obviously each patient belongs to only one condition) as "collinear". In the introduction, they even mention that fixed effect models will result in an ill-posed optimization that cannot be solved. This is not the case: this type of design is known in the experimental design literature as a “split-plot design” and many classical solutions exist to analyze such designs using fixed effects. Of course, computationally, this becomes challenging when the number of patients increases, and it becomes harder to generalize beyond the patients observed in the experiment. This is a point that the authors can make in favor of their approach.

2. Both in their model specification and in the simulation studies, the authors assume that the cell type labels are known. While this is common practice in the scRNA-seq field, it can lead to artificially inflating the significance of DE tests (see e.g., [1] and [2]). While I don’t expect the authors to tackle this problem in this paper, they should acknowledge this limitation and discuss it in the introduction and/or discussion sections.

3. I found it confusing that Figure 2A presents the speedup instead of the actual computational time of the three compared approaches. I would suggest presenting the actual time in the main figure and the speedup in the supplementary.

4. Panel 2C and 2D (and the supplementary analogs) are not a good visualization of the simulation results. The y axes of the density plots are too small to understand the extent of the departure from the expected uniform distribution for the most interesting values (the interval [0, 0.05]). Qq-plots or histograms seem like a better visualization for Fig. 2C. Moreover, Figure

2D would be more informative in the context of the (lack of) type I error control. I find that a very good visualization in this context is the TPR/FDR curve computed e.g. by the iCOBRA R package [3], which can make it clearer what is the power of a given method for a nominal FDR threshold.

5. While it is standard practice to use pseudo-bulk approaches for DE analysis in what the authors call the “patient-wise” setting. I have not seen such an analysis in the “cell-wise” setting. Since it’s not standard, the authors should describe in more detail how they perform it (is it comparing one pseudo-bulk sample for each patient-cell-type combination across cell types?). Moreover, they should acknowledge that this is not a commonly used technique. The authors should add a straightforward limma (or edgeR, see below) analysis (at the cell level, without accounting for patient differences) to their benchmark, as this is common practice.

6. Choice of competing methods: the authors acknowledge that edgeR and DESeq2 are the most common choices for DE analysis in RNA-seq. Yet, these methods are missing from their benchmark. I would like to see the results of a pseudo-bulk approach based on edgeR (or DESeq2) in addition to the one based on limma.

7. Limma analysis: one of the advantages of the limma package is that it allows to model intra-sample correlations (see the `duplicateCorrelation` function and its manual page). The authors should compare their sandwich estimator approach with the limma analysis done at the single-cell level but with intra-patient correlations estimated by the `duplicateCorrelation` function.

8. In their analyses, the authors compare the different approaches based on their ability to recover what the authors refer to as “ground-truth cell types”. However, I believe that in all the datasets used in the paper, the cell type labels are not in fact “ground truth”, but data-driven labels inferred from the data itself. In addition to the double-dipping issue mentioned above, this makes the analyses presented in Figure 3 very weak. Some datasets exist with some ground truth information, e.g., cell-line mixing [4,5]. CITE-seq datasets might also provide a good testing ground, as the authors could show which method can find markers that are also found in the antibody part of the dataset.

9. In the analysis presented in Figure 4, are the authors looking at DE genes between young and old that are common among all cell types? Does it mean that they are creating a single pseudo-bulk sample for each patient across all cell types? If this is the case, I would suggest to look at cell types separately, as this is more aligned with the typical analysis that practitioners would perform. This can be done with interaction terms in the authors’ approach and would allow to directly test whether some genes are DE in type 1 or type 2 myonuclei (or both).

10. From Figure 4A, it looks like there may be a confounding between cell type and condition as it appears that more type 1 myonuclei are in old samples and more type 2 myonuclei in young samples. Is this the case? What would change if the data were subsampled to create a balanced setting?

11. Figure 4A was confusing for me, and it took me a while to figure out that the blue and gold points are a zoom in of the red and green cluster. Can you make it more explicit by adding connecting lines or by making it two separate panels?

12. I tried the R package, and it works well on my system. I commend the authors for providing easy-to-use open-source code and clear documentation. However, for the long-term maintenance, versioning, and testing of the package, I would recommend submitting the package to Bioconductor or CRAN. At the very least, to ensure reproducibility, the authors should share on Zenodo (or similar service with DOI) a frozen version of the package that corresponds to the version used for the analyses presented in the paper.

13. It is not clear from the Methods section that the scaling factor s is fixed prior to model fitting. Indeed s appears only in equation (3) and is missing from the rest of the exposition. Please make this explicit.

14. \Sigma_\beta is never defined. I suspect it’s the variance matrix related to the estimated beta vector, but this is never explicitly stated. Perhaps explicitly using the \hat\beta notation to refer to the estimated parameters and calling it \Sigma_\hat\beta would help.

15. The authors show the scalability of their approach for increasing numbers of genes and cells. However, given that their approach involves modeling intra-patient correlations, the authors should also look at scalability in the number of patients (for a given total number of cells), and in the number of covariates (features in the authors' notation).

16. The authors use a large number of high-end GPUs in their benchmark. I suspect that many practitioners will have access to fewer resources: perhaps a single GPU, perhaps with only a few GB of VRAM. It would be useful to compare the performance of their method in such scenarios, a “small” GPU with 16GB of VRAM, and a “medium” GPU with 48GB of VRAM, in addition to their multi-GPU setting.

References:
[1] Neufeld, A., Gao, L. L., Popp, J., Battle, A., & Witten, D. (2024). Inference after latent variable estimation for single-cell RNA sequencing data. Biostatistics, 25(1), 270-287.
[2] Gao, L. L., Bien, J., & Witten, D. (2024). Selective inference for hierarchical clustering. Journal of the American Statistical Association, 119(545), 332-342.
[3] Soneson C, Robinson MD (2016). “iCOBRA: open, reproducible, standardized and live method benchmarking.” Nature

Methods, 13(4), 283.
[4] Tian, Luyi, et al. (2019) Benchmarking single cell RNA-sequencing analysis pipelines using mixture control experiments. Nature Methods, 16(6), 479-487.
[5] Arigoni, M., Ratto, M. L., Riccardo, F., Balmas, E., Calogero, L., Cordero, F., et al. (2024). A single cell RNAseq benchmark experiment embedding “controlled” cancer heterogeneity. Scientific Data, 11(1), 159.

(Remarks on code availability)
While I did not extensively reviewed the code, I did successfully install and used the package on my system (without a GPU). The package is well-documented and easy to use.

Version 1:

Reviewer comments:

Reviewer #1

(Remarks to the Author)
I appreciate the authors' dedication to this revision. The authors addressed my comments well.

I have one more comment. In Discussion, the authors state that “Moreover, for the first time, our approach is the only that can analyse datasets with over 10 million cells and thousands of genes efficiently.”

I find the recent pseudobulk-based DE tool “dreamlet” attractive when analyzing millions of cells, as it takes only a few minutes for 1M cells. Thus, the above sentence could be overstatement. Although superior accuracy of devil over pseudobulk methods are shown well in Fig 2, such advantage could be rather reduced when analyzing millions of cells. Is there computational bottleneck to apply pseudobulk methods to 10M cells? Pros and cons between pseudobulk method like dreamlet and devil need to be stated to clearly guide users when and why to use this tool.

Other minor comments:
The numbers of cells used in each study need to be stated in legends of Suppl Fig 7~10.
Line 218 needs revision
What happens to MAST curves in Fig. 2D?
Legend of Fig 2CD needs revision
Ref 31. Preprint

(Remarks on code availability)

Reviewer #2

(Remarks to the Author)
I appreciate the authors great improvements of the manuscript and have only a few remaining comments:
Figure 2: Figure 2 C and D - benchmarking: the different models are not so easily distinguished or out of frame, a comprehensive overview (maybe in the supplement) might help to show the data more fully. Additionally, while the extended figure clearly shows the speed-up of devil compared to NEBULA, I think it is important to highlight this in Figure 2A, as in other metrics (MCC and TPR QQ-Plots), NEBULA shows the highest similarity to devil. Thus, to strengthen the advantages of devil, I would more clearly highlight the comparison and the benefits of devil.
P.9 l.244 possible typo: 5 markers
Extended Figure 3: Please adjust the heatmap to a colour scheme that is not red-green (e.g. use blue instead of green)
P.13 l.357 typo/ missing adjustment: 10 million cells
Lastly to make this upgraded method more accessible to potential users, I think it should be noted in the manuscript, that this method is available as an R package. Additionally computational requirements and R versions should be listed as is common when publishing a new package.

(Remarks on code availability)

Reviewer #3

(Remarks to the Author)
The authors thoroughly addressed all the comments in my previous review and I am satisfied with the current version of the manuscript.

I only have the following minor points that are needed to improve the clarity of the presentation:

1. Please specify in the text the meaning of the multiplication operator in equation 1, as it is not a standard notation in bioinformatics.

2. In responding to a comment by another reviewer, the authors have implemented a new approach for the estimation of the dispersion parameter based on the method of moments. If I understand correctly all the results presented in this version of the manuscript are obtained using this approach. It would be useful for a reader to show in a supplementary figure or table that the two estimators achieve qualitatively the same results in practice.

3. In Section 4.9 the authors state that "the wall-clock time scale with the number of GPUs, reaching a maximum speedup of 5.2 using 8 GPUs." The speedup of 5.2 is compared to what? A single GPU? Please clarify.

(Remarks on code availability)
While it is straightforward to install the package without GPU support, unsurprisingly, I struggeled installing with CUDA support.

The authors provide good guidelines in two dedicated vignettes, but I suggest to add this content (or a link to the vignettes) in the README, as most users would expect to find detailed installation instructions there.

Reviewer #4

(Remarks to the Author)
I co-reviewed this manuscript with one of the reviewers who provided the listed reports. This is part of the Nature Communications initiative to facilitate training in peer review and to provide appropriate recognition for Early Career Researchers who co-review manuscripts.

(Remarks on code availability)

Version 2:

Reviewer comments:

Reviewer #1

(Remarks to the Author)
The authors addressed all my comments.

(Remarks on code availability)

Reviewer #2

(Remarks to the Author)
The authors addressed the comments well and I am satisfied with the current version of the manuscript.

(Remarks on code availability)

Reviewer #3

(Remarks to the Author)
The authors satisfactorily addressed my remaining comments. I congratulate the authors on a nice paper.

(Remarks on code availability)

Reviewer #4

(Remarks to the Author)
I co-reviewed this manuscript with one of the reviewers who provided the listed reports. This is part of the Nature Communications initiative to facilitate training in peer review and to provide appropriate recognition for Early Career Researchers who co-review manuscripts.

(Remarks on code availability)

Open Access This Peer Review File is licensed under a Creative Commons Attribution 4.0 International License, which permits use, sharing, adaptation, distribution and reproduction in any medium or format, as long as you give appropriate credit to the original author(s) and the source, provide a link to the Creative Commons license, and indicate if changes were made.
In cases where reviewers are anonymous, credit should be given to 'Anonymous Referee' and the source.
The images or other third party material in this Peer Review File are included in the article's Creative Commons license, unless indicated otherwise in a credit line to the material. If material is not included in the article's Creative Commons license and your intended use is not permitted by statutory regulation or exceeds the permitted use, you will need to obtain permission directly from the copyright holder.
To view a copy of this license, visit https://creativecommons.org/licenses/by/4.0/

RESPONSE TO REVIEWERS' COMMENTS

Replies in red, comments in bold.

Reviewer #1 (Remarks to the Author):

The authors present devil, a novel Bayesian framework for single-cell differential expression (scDE) testing that is both statistically rigorous and computationally scalable, capable of handling datasets with over 10 million cells across multiple patients. The method addresses critical limitations in existing approaches: conventional methods incorrectly assume cell-level independence, while pseudobulk method ignores cell-to-cell variation. The proposed method uses GPU-based variational inference for scalable estimation of regression coefficients. It also uses a clustered sandwich covariance approach to address dependence of cells within patients, thereby avoiding explosion of subject-specific parameters as seen in the mixed effect model. Overall, the authors provide a timely and important contribution to the field of scalable and accurate scDE analysis.

Comments for the authors:

1. It would be interesting to include comparisons with commonly used scDE tools such as FindMarkers (Seurat, Wilcoxon test) and MAST where feasible within a reasonable runtime threshold. This would give users practical intuition about when devil should be preferred over these widely adopted methods.

Thank you for this suggestion. In response to this and related reviewer feedback (Point 5 and 6 by Reviewer 3), we substantially expanded the set of methods evaluated in both the cell-wise and patient-wise benchmarks, with the aim of providing clearer guidance on which tools are most appropriate under different experimental designs. The evaluated methods are now reported in Supplementary Table 2.

In this revised manuscript we added comparisons with MAST at the single-cell level (in the main text), and with the Seurat Wilcoxon rank-sum test (in the Supplementary Text). These additions allow direct comparison with widely used scDE approaches and help contextualise when devil should be preferred over standard methods.

In brief, results show that single-cell methods that treat cells as independent observations (including glmGamPoi, MAST, and the Seurat Wilcoxon test) frequently

exhibit inflated p-values and reduced accuracy. We have added a commentary on these results in Sec. 2.2, modified Figure 2 and Extended Figure 1 to accommodate additional methods, as well as Supplementary Figure 7-10 to accommodate all tested methods.

2. In cell-wise design (e.g., cell type marker identification), treating batch effects is essential in atlas-scale scDE. How does devil account for batch effects? A dedicated benchmark, either through simulations or real scRNA-seq datasets with known batch structure, would clarify devil's robustness in these scenarios.

Thank you for raising this important point. To address batch effects in cell-wise designs, we conducted a dedicated robustness analysis (discussed in sec 2.2 and described in the section Evaluation of batch-effect control of the Online Methods) under multi-batch experimental settings.

Specifically, we evaluated devil across three dimensions:

- dataset scale,
- batch-effect strength, and
- true differential expression effect size.

We compared three analysis strategies:

- (i) no batch covariates and no clustering,
- (ii) patient-clustered (robust) inference without batch covariates, and
- (iii) explicit batch covariates combined with patient-clustered inference.

This benchmark demonstrates that patient-clustered inference substantially mitigates the impact of batch effects through robustness to model misspecification, even when batch structure is not explicitly modelled. However, as expected, explicitly including batch covariates remains optimal under strong batch effects.

These results are now described in Section 2.2, with the inclusion of new Supplementary Figures 5 and 6.

3. For broader adoption, the authors could provide additional test examples illustrating different experimental designs and application settings (with and without GPU acceleration). A more detailed user manual or tutorials on the GitHub repository would also be highly valuable.

To better illustrate devil's flexibility across different experimental designs, and in response to related reviewer feedback (points 9 by Reviewer 3), we expanded the skeletal muscle case study by introducing additional interaction terms in the design matrix. This extension requires simultaneously modeling condition-specific effects within predefined cellular sub-population while preserving valid inference under

patient-level clustering and unequal cell numbers. This extended analysis enables the detection of functionally relevant, condition-specific biological programs within distinct cellular populations, rather than averaging effects across heterogeneous cell types. These results are now discussed in Section 2.4, with an updated Figure 4, an expanded Extended Figure 3, and the addition of Supplementary Figures 14–18.

The new manuscript presents an extended analysis explicitly demonstrates devil's ability to test condition-specific effects (e.g., ageing) within defined cellular sub-populations.

In addition, we substantially enriched the documentation and tutorials available on the GitHub repository. These now include:

- detailed user manuals describing core implementation choices (including CPU and GPU execution)
- worked examples covering a range of experimental designs. In brief, these include design matrices with multiple columns encoding the same biological factor, such as cell-type–specific effects; multi-covariate designs combining distinct experimental variables (e.g., stimulation condition and age group); designs with continuous covariates, illustrated through time-resolved analyses using days after start of experiment.

Together, these additions provide clearer guidance on practical usage and facilitate broader adoption of devil.

4. In Fig. 2E (third panel), the MCC of glmGamPoi is strikingly low. Is this entirely attributable to its failure in false-positive control, or might there be additional factors contributing to this result? A deeper discussion would help readers interpret this discrepancy.

Thank you for highlighting this point. The markedly low MCC of glmGamPoi in the patient-wise setting is primarily driven by overly confident p-values, which lead to insufficient false-positive control and a substantially inflated false positive rate. This, consequently, severely degrades MCC performance.

We have clarified this mechanism explicitly in the revised text (see sec 2.2) and further illustrated it with additional visualisations highlighting false-discovery-rate (see new Fig. 2C,D).

Reviewer #2 (Remarks to the Author):

The authors present devil, a framework for scDE testing that accounts for statistical dependence between individual samples. They demonstrate that their method performs on par or better with current state-of-the-art

approaches and offers a significant benefit in terms of computational performance. While the manuscript is of relevance and matches the scope of Nat Commun, some changes in the manuscript are necessary and major concerns are related to the code.

Comments for the authors:

p. 4, l. 62: the notion of a design matrix X suggests that the considered covariates may only represent discrete levels (e.g., experimental conditions). Please clarify whether the approach can be extended towards continuous covariates (e.g., concentrations, age, …) and state this (possible) limitation in the discussion.

Thank you for pointing this out. Although we did not state this explicitly in the original manuscript, the design matrix formulation in devil naturally supports both discrete and continuous covariates, including variables such as age or concentrations. We have now clarified this explicitly in the revised text (see sec 2.1).

In addition, to make this feature more accessible to users, we expanded the documentation of the R package to include a dedicated worked example demonstrating the use of continuous covariates.

p. 4 l. 98: CPU and GPU vendor and model should be named in the main text, or at least include a reference to the supplementary table. Compiler version, CUDA version, R version etc. should be detailed in the appendix / supplements for reproducibility.

We thank the reviewer for highlighting this important point regarding reproducibility. In the revised manuscript we include a detailed description of the computational environment.

Specifically, we have added a new *Supplementary Table 4*, which lists the hardware specifications (server/CPU/GPU vendor and model) and the complete software stack (OS, Compiler, CUDA, R, and BLAS versions).

We have also updated *Section 4.9 GPU Acceleration* in the text to explicitly reference this table and to enrich the computational environment description.

To ensure reproducibility, upon acceptance of the manuscript we will freeze both the devil package and the analysis workflow corresponding to the results presented in this study and deposit them on Zenodo, providing permanent, versioned

snapshots of the software and all analysis scripts. During the review process, the package and reproducibility materials are openly available in a public GitHub repository, with the links to them included in the manuscript.

p.4 l. 102: Does the high memory usage of glmGamPoi arise from the concept / methodology, or from a sub-optimal implementation? In case of the latter, this should be mentioned in the text for fair comparison.

Thank you for this important question. To better understand the origin of the observed memory differences, we profiled glmGamPoi using *bench::mark* to analyze its memory behavior.

Our analysis indicates that the increased memory footprint of glmGamPoi arises primarily from its computational strategy. Specifically, glmGamPoi operates on full gene-by-cell matrices during model fitting without gene-wise parallelization. This approach requires maintaining large matrices in memory throughout the computation, with memory allocations scaling linearly with gene count (O(genes × cells)). This design choice increases both peak memory usage and memory bandwidth consumption, which in turn limits scalability at large dataset sizes. In contrast, gene-wise streaming approaches, like the one we employ, process individual gene vectors sequentially, maintaining constant memory overhead independent of gene number.

Our analysis revealed that the number of memory allocations for matrices scales linearly with the number of genes, resulting in a significantly larger memory footprint and increased bandwidth consumption.

p. 4 l. 103: The extrapolation method should be explained precisely. Is it a linear extrapolation, nonlinear etc.?

Thank you for pointing this out. In the revised analysis, we removed the need for the extrapolation steps..

Specifically, we increased the available computational resources for all methods (up to approximately 1 TB of memory), allowing each method to complete its execution without relying on estimated runtime or memory usage.

As a result, all reported timing and memory measurements are now directly observed rather than extrapolated, and the extrapolation procedure has been removed from both the analysis and the manuscript.

p. 7 l. 107: Please provide confirmation that devil yields the exact same p-values for the CPU and GPU implementation.

Thank you for this question. We now explicitly show that the CPU and GPU implementations of devil yield perfectly correlated results, both in terms of inferred model parameters and associated p-values (section 2.2, see Extended Figure 1A,B and Supplementary Figure 2).

Due to expected numerical differences arising from floating-point arithmetic and hardware-specific optimisations, the results are not numerically identical at machine precision. However, we demonstrate that these differences have no practical impact: *downstream statistical conclusions are identical between the two implementations*.

p. 7, l. 112: Why did the authors choose not to optimize the MLE inference before submission of the manuscript? Please elaborate in the manuscript how such optimization would be implemented.

In the original submission, we did not optimize the MLE-based inference because it relied on special functions (e.g., *digamma* and *trigamma*) that are difficult to accelerate efficiently on GPUs and limit scalable parallelisation. As a result, we considered that further optimisation of the MLE path would have yielded limited practical gains in large-scale settings.

However, following this feedback, we now explored alternative estimation strategies that are naturally suited to GPU execution. This led us to introduce a *method-of-moments* (MoM) estimator (see sec. 2.1, 4.1 and Method-of-moments estimator paragraph of Supplementary Text), which avoids special functions, is highly parallelisable, and achieves substantial speedups on GPU architectures. The MoM is based on the idea of estimating dispersion by matching the empirical variance of residuals to the model-implied variance. This new implementation, along with other smaller optimizations of the code, allowed us to reach a gain of up to 40x over glmGamPoi for large datasets.

The results in the revised manuscript are now based on the new MoM estimator, but for completeness, devil continues to support the original MLE-based inference (which remains available but is substantially slower).

p. 7, l. 135-144: Do the reported standard deviations refer to the simulation design reported in ll. 506? If so, please include a reference in the text

We're sorry for the lack of clarity. We add a reference in the results section pointing to the Online methods description of the simulation (see sec 2.2 now referring to the *Simulation study of experimental design structure* paragraph of the Online Methods).

p. 12, l. 238: typo implementedd

Fixed.

p. 21, l. 611: Please perform a strong scaling experiment to demonstrate the scalability of devil and include it in the supplementary information. Based on the results in Suppl. Fig. 1B it would be expected that the strong scaling efficiency is limited by the CPU-part of the code.

We thank the reviewer for this suggestion. In response, we performed a strong scaling analysis of GPU implementation by fixing the total problem size across a grid of representative dataset configurations, spanning combinations of 10k, 100k, and 1M cells with 1k, 5k, and 10k genes, and increasing the number of GPUs.

In this large scale-test we achieved a speedup of 5.2x on 8 GPUs**.** Regarding the reviewer's observation on CPU limitations, in the revised version we significantly reduced CPU overhead by introducing the new Method of Moments–based initialization, which substantially improved strong-scaling performance.

As a result, the primary scalability penalty shifted away from the CPU and toward load imbalance across GPUs, arising from heterogeneous gene convergence rates.

These results are now included in the Supplementary Figure 2.

Figure 3E: Why does the accuracy not improve further when adding more than ~100 markers?

Thank you for this question. We now clarify this behaviour in the manuscript (see sec 2.3). Briefly, as the number of markers increases beyond approximately 100, gene sets become progressively less specific to the underlying biological signal. This introduces ambiguity in the classification task, leading to a gradual decline and convergence of accuracy across methods.

Extended Figure 2A: Please add annotations for the cell type to the legend.

Thank you for noticing this. We have now added cell-type annotations to the legend in the revised figure (now Extended Fig. 3A).

Reviewer #2 (Remarks on code availability):

Comments on Code

- **The Github repository does not contain any unit tests for any of the branches (CPU version, GPU version, Python bindings). It is strictly necessary to add tests where possible before acceptance of the manuscript.**

Thank you for highlighting this point. In response to this and related reviewer comments, we have substantially strengthened the software by adding a comprehensive suite of unit tests, we expanded the documentation and tutorials to improve usability and reproducibility. In particular, now we have:

- detailed user manuals describing core implementation choices (including CPU and GPU execution)
- worked examples covering a range of experimental designs. In brief, these include design matrices with multiple columns encoding the same biological factor, such as cell-type–specific effects; multi-covariate designs combining distinct experimental variables (e.g., stimulation condition and age group); designs with continuous covariates, illustrated through time-resolved analyses using days after start of experiment.

These additions make the package aligned with established standards such as Bioconductor.

• The GPU implementation in the devel branch appears to be incomplete. For example, the function "beta_fit_gpu_external" is used in beta.cpp but is not defined in the repository. Did I miss something? How can I inspect the code for the GPU implementation? Since the GPU version is central to the manuscript, it must be available and installable via GitHub.

Thank you for noticing this. The GPU code was originally developed as a separate library and maintained in a dedicated repository, with the sources fetched and compiled during the configuration step.

To avoid any concern, we now have moved the GPU code into the main repository to make it fully accessible, easier to inspect, and simpler to maintain.

• As a minor comment, the README.md should be updated for the Python branch (if it is considered stable, which I assume since it is mentioned on the main branch)

Reviewer #3 (Remarks to the Author):

The authors present a novel approach for the differential expression analysis of single-cell RNA-seq data that takes into account the hierarchical design of multi-sample datasets and that is scalable to millions of cells. The approach

is grounded in sound statistical approaches (sandwich estimator, mean-field variational inference) and is rendered attractive by the GPU implementation that makes it ultra scalable. The implementation in R allows users familiar with typical differential expression packages, such as edgeR and DESeq2, to easily adapt their pipeline. Overall, the proposed method seems a good addition to the tools available for differential expression in large, multi-sample datasets.

However, I have several major points to be addressed by the authors.

Comments for the authors:

1. The authors refer to a design in which cells are collected from multiple patients per condition (in which obviously each patient belongs to only one condition) as "collinear". In the introduction, they even mention that fixed effect models will result in an ill-posed optimization that cannot be solved. This is not the case: this type of design is known in the experimental design literature as a “split-plot design” and many classical solutions exist to analyze such designs using fixed effects. Of course, computationally, this becomes challenging when the number of patients increases, and it becomes harder to generalize beyond the patients observed in the experiment. This is a point that the authors can make in favor of their approach.

Thank you for this clarification. Our description was indeed not precise, and we agree that this experimental setup corresponds to a classical split-plot design and that fixed-effect formulations are statistically valid in this context.

We have revised the manuscript to reflect this more precisely and to avoid any implication that such designs are inherently ill-posed (see sec. 1). In the revised text, we clarify that they can become increasingly cumbersome in practice as the number of patients grows, particularly when patient identity is closely aligned with experimental conditions.

2. Both in their model specification and in the simulation studies, the authors assume that the cell type labels are known. While this is common practice in the scRNA-seq field, it can lead to artificially inflating the significance of DE tests (see e.g., [1] and [2]). While I don’t expect the authors to tackle this problem in this paper, they should acknowledge this limitation and discuss it in the introduction and/or discussion sections.

Thank you for this comment.

We now explicitly acknowledge this limitation in the Discussion, noting that our analyses assume fixed cell-type annotations and that uncertainty in cell labelling (e.g when derived from clustering) can inflate DE significance.

As you suggest, we clarify that addressing label uncertainty lies beyond the scope of this work and represents an important direction for future extensions of devil.

3. I found it confusing that Figure 2A presents the speedup instead of the actual computational time of the three compared approaches. I would suggest presenting the actual time in the main figure and the speedup in the supplementary.

Thank you for this suggestion. We changed Fig. 2A to display the actual computational time and moved the corresponding speedup analysis to Supplementary Figure 1.

4. Panel 2C and 2D (and the supplementary analogs) are not a good visualization of the simulation results. The y axes of the density plots are too small to understand the extent of the departure from the expected uniform distribution for the most interesting values (the interval [0, 0.05]). Qq-plots or histograms seem like a better visualization for Fig. 2C. Moreover, Figure 2D would be more informative in the context of the (lack of) type I error control. I find that a very good visualization in this context is the TPR/FDR curve computed e.g. by the iCOBRA R package [3], which can make it clearer what is the power of a given method for a nominal FDR threshold.

We revised the visualisation of the simulation results as suggested. These changes should substantially improve the interpretability of the simulation results.

5. While it is standard practice to use pseudo-bulk approaches for DE analysis in what the authors call the “patient-wise” setting. I have not seen such an analysis in the “cell-wise” setting. Since it’s not standard, the authors should describe in more detail how they perform it (is it comparing one pseudo-bulk sample for each patient-cell-type combination across cell types?). Moreover, they should acknowledge that this is not a commonly used technique. The authors should add a straightforward limma (or edgeR, see below) analysis (at the cell level, without accounting for patient differences) to their benchmark, as this is common practice.

Thank you for highlighting this point.

We have clarified the pseudo-bulk construction procedure in the revised manuscript (Section 2.1). Specifically, in both the patient-wise and cell-wise settings,

pseudo-bulk counts are obtained by aggregating cells within each (patient, condition) group.

Additionally, we included a straightforward single-cell analysis using limma at the cell level, which is now part of the benchmark. Results show that this approach performs worse than dedicated single-cell methods and also worse than limma applied to pseudo-bulk data, consistent with the known limitations of linear modeling of log-transformed counts when dealing with very low or zero counts common in single-cell data.

6. Choice of competing methods: the authors acknowledge that edgeR and DESeq2 are the most common choices for DE analysis in RNA-seq. Yet, these methods are missing from their benchmark. I would like to see the results of a pseudo-bulk approach based on edgeR (or DESeq2) in addition to the one based on limma.

Thank you for this suggestion.

In response to this and feedback from Reviewer 1, we substantially expanded the set of methods evaluated in both the cell-wise and patient-wise benchmarks, with the aim of providing clearer guidance on method choice across experimental designs.

We discuss this in detail in response to point 1, Reviewer 1 (specifically addressing your point, we added a pseudo-bulk differential expression analysis based on edgeR, which is now included in the main text). Similarly to the other pseudo-bulk method testes (i.e. limma), edgeR consistently underperformed in the cell-wise settings due to loss of within-patient information.

7. Limma analysis: one of the advantages of the limma package is that it allows to model intra-sample correlations (see the \`duplicateCorrelation\` function and its manual page). The authors should compare their sandwich estimator approach with the limma analysis done at the single-cell level but with intra-patient correlations estimated by the \`duplicateCorrelation\` function.

Following this suggestion, we added a comparison using limma at the single-cell level with intra-patient correlations estimated via the “*duplicateCorrelation*” procedure.

While effective for designs with a limited number of correlated replicates, this approach performs poorly at single-cell scale in terms of both computational cost and statistical calibration, reflecting practical limitations of “*duplicateCorrelation*” in single-cell analyses.

In particular, we observed that limma with “*duplicateCorrelation*” achieved performance comparable to, but not consistently better than, limma applied to single cells without correlation modeling, with results varying across datasets and experimental settings. Moreover, the computational overhead was substantial, making it by far the slowest method in our benchmark (up to two orders of magnitude slower than devil). These results are now reported in Supplementary Figures 7 to 10.

8. In their analyses, the authors compare the different approaches based on their ability to recover what the authors refer to as “ground-truth cell types”. However, I believe that in all the datasets used in the paper, the cell type labels are not in fact “ground truth”, but data-driven labels inferred from the data itself. In addition to the double-dipping issue mentioned above, this makes the analyses presented in Figure 3 very weak. Some datasets exist with some ground truth information, e.g., cell-line mixing [4,5]. CITE-seq datasets might also provide a good testing ground, as the authors could show which method can find markers that are also found in the antibody part of the dataset.

We thank the reviewer for this important clarification.

We agree that in most scRNA-seq datasets, cell type labels are data-driven rather than true ground truth, which can introduce circularity. To address this concern, we revised the analysis in two ways.

First, we repeated the analysis using the popular dataset from Hao et al. (“*Integrated analysis of multimodal single-cell data*”, Cell, Volume 184, Issue 13, 3573 - 3587.e29), in which cell type annotations are informed by both RNA expression and CITE-seq antibody measurements. This substantially reduces the dependence of labels on RNA alone and mitigates circularity.

Second, we included an additional benchmark based on a dataset of 10 purified immune cell types from Fu et al. (“*A comparison of scRNA-seq annotation methods based on experimentally labeled immune cell subtype dataset*”, Briefings in Bioinformatics, Volume 25, Issue 5, September 2024, bbae392,), where cell identities can be treated as ground truth. This additional analysis is now discussed in section 2.3 and described in detail in the Online Methods (paragraph *Marker gene recovery analysis*). The results can also be visualized in Extended Figure 2. Briefly, in this setting, we observe again that accounting for cell-to-cell correlation improves marker gene detection. In fact, using method-specific p-values as a binary classifier of marker versus non-marker genes, we obtained average AUCs of 0.743 and 0.738 for devil and NEBULA, respectively, while glmGamPoi achieved a lower AUC of 0.691. These results further support the conclusion that explicitly modeling correlation between cells leads to more robust differential expression inference.

9. In the analysis presented in Figure 4, are the authors looking at DE genes between young and old that are common among all cell types? Does it mean that they are creating a single pseudo-bulk sample for each patient across all cell types? If this is the case, I would suggest to look at cell types separately, as this is more aligned with the typical analysis that practitioners would perform. This can be done with interaction terms in the authors' approach and would allow to directly test whether some genes are DE in type 1 or type 2 myonuclei (or both).

Thank you for this clarification.

In Fig. 4, differential expression is performed at the single-cell level rather than via pseudo-bulking, and genes are not restricted to those shared across all cell types.

In response to your suggestion, we extended the analysis by explicitly including an interaction term between age and cell type in the design matrix. This allows us to directly test ageing-associated effects separately in Type I and Type II myonuclei (and to assess shared versus subtype-specific responses).

These results are now reported in the revised manuscript. In brief, we observe that ageing-associated transcriptional changes are largely cell-type–specific: Type I myonuclei are dominated by structural and sarcomeric remodelling programs, whereas Type II myonuclei show stronger translational, immune, and stress-response signatures.

10. From Figure 4A, it looks like there may be a confounding between cell type and condition as it appears that more type 1 myonuclei are in old samples and more type 2 myonuclei in young samples. Is this the case? What would change if the data were subsampled to create a balanced setting?

Thank you for raising this point. To assess the potential confounding between age and cell type, we performed an additional analysis in which the dataset was subsampled to create a balanced design, with equal numbers of cells for each group (where a group is defined as age and cell-type combination, see sec 2.4).

This new analysis shows that the main conclusions are robust and that devil is the method whose downstream results are least affected by subsampling (Extended Data Fig. 3C).

11. Figure 4A was confusing for me, and it took me a while to figure out that the blue and gold points are a zoom in of the red and green cluster. Can you

make it more explicit by adding connecting lines or by making it two separate panels?

To improve clarity, we revised Fig. 4A by splitting it into two separate panels, making the zoomed-in view explicit.

12. I tried the R package, and it works well on my system. I commend the authors for providing easy-to-use open-source code and clear documentation. However, for the long-term maintenance, versioning, and testing of the package, I would recommend submitting the package to Bioconductor or CRAN. At the very least, to ensure reproducibility, the authors should share on Zenodo (or similar service with DOI) a frozen version of the package that corresponds to the version used for the analyses presented in the paper.

Thank you for this suggestion and for testing the package.

To ensure reproducibility, upon acceptance of the manuscript we will freeze both the devil package and the analysis workflow corresponding to the results presented in this study and deposit them on Zenodo, providing permanent, versioned snapshots of the software and all analysis scripts. During the review process, the package and reproducibility materials are openly available in a public GitHub repository, with the links to them included in the manuscript.

In parallel, we are working toward submitting the package to Bioconductor for long-term maintenance.

13. It is not clear from the Methods section that the scaling factor is fixed prior to model fitting. Indeed s appears only in equation (3) and is missing from the rest of the exposition. Please make this explicit.

Thank you for pointing this out. We have clarified in sec 2.1 that the scaling factor s is fixed prior to model fitting and added references to commonly used size-factor normalisation strategies.

14. \Sigma_\beta is never defined. I suspect it's the variance matrix related to the estimated beta vector, but this is never explicitly stated. Perhaps explicitly using the \hat\beta notation to refer to the estimated parameters and calling it \Sigma_\hat\beta would help.

In our notation, \Sigma_\beta denotes the covariance associated with a given parameter vector \beta in a general sense.

Depending on the context, \beta may correspond to initial, intermediate, or converged (estimated) values during optimization, and we intentionally use a single notation to cover these cases.

While we agree that explicitly introducing \hat{\beta} is a valid alternative, we chose to retain the more compact notation to keep the presentation simple. We have nevertheless revisited the surrounding text to ensure that the role of \Sigma_\beta is clearly stated at its first use (see sec 2.1).

15. The authors show the scalability of their approach for increasing numbers of genes and cells. However, given that their approach involves modeling intra-patient correlations, the authors should also look at scalability in the number of patients (for a given total number of cells), and in the number of covariates (features in the authors' notation).

Thank you for this suggestion. We have extended the scalability analysis (see sec 2.2) to explicitly assess performance as a function of both the number of patients and the number of covariates, while keeping the total number of cells fixed.

These additional benchmarks are now discussed in sec 2.2 and described in the Online Methods ("Scaling across patients and features"). Results show that increasing the number of patients substantially impacts the runtime of NEBULA, reflecting the cost of modeling subject-specific random effects, whereas devil maintains stable scaling. In contrast, scaling with respect to the number of covariates affects all methods similarly, with relative speedups remaining approximately constant as feature dimensionality increases.

16. The authors use a large number of high-end GPUs in their benchmark. I suspect that many practitioners will have access to fewer resources: perhaps a single GPU, perhaps with only a few GB of VRAM. It would be useful to compare the performance of their method in such scenarios, a "small" GPU with 16GB of VRAM, and a "medium" GPU with 48GB of VRAM, in addition to their multi-GPU setting.

Thank you for this comment; we agree that access to large multi-GPU and high-end systems is not a trivial requirement.

To address this concern, we expanded our GPU evaluation to explicitly analyze how *devil* scales with batch size and number of devices, with particular attention to VRAM requirements.

These results show that *devil* does not require high-end or multi-GPU setups. In particular, our analysis shows *devil* provides substantial acceleration even on

standard single-GPU systems, while multi-GPU setups primarily providing additional, but not essential, acceleration.

As shown in Supplementary Figure 2, a batch size of 100 enables inference on 1E6 cells using less than 6 GB of GPU memory. Moreover, Supplementary Table 5 shows that good performance is achieved using a single GPU, with runtimes on the order of tens to hundreds of seconds. Although these runtimes were measured using 8 GPUs, the scalability analysis in Supplementary Figure 2 indicates that using a single GPU would result in a runtime at most about five times longer, given a maximum observed speedup of approximately 8.

References:
[1] Neufeld, A., Gao, L. L., Popp, J., Battle, A., & Witten, D. (2024). Inference after latent variable estimation for single-cell RNA sequencing data. Biostatistics, 25(1), 270-287.
[2] Gao, L. L., Bien, J., & Witten, D. (2024). Selective inference for hierarchical clustering. Journal of the American Statistical Association, 119(545), 332-342.
[3] Soneson C, Robinson MD (2016). "iCOBRA: open, reproducible, standardizedand live method benchmarking." Nature Methods, 13(4), 283.
[4] Tian, Luyi, et al. (2019) Benchmarking single cell RNA-sequencing analysis pipelines using mixture control experiments. Nature Methods, 16(6), 479-487.
[5] Arigoni, M., Ratto, M. L., Riccardo, F., Balmas, E., Calogero, L., Cordero, F., et al. (2024). A single cell RNAseq benchmark experiment embedding "controlled" cancer heterogeneity. Scientific Data, 11(1), 159.

Reviewer #3 (Remarks on code availability):

While I did not extensively reviewed the code, I did successfully install and used the package on my system (without a GPU). The package is well-documented and easy to use.

RESPONSE TO REVIEWERS' COMMENTS

We thank the reviewers for their positive feedback and constructive critiques, which were extremely useful in strengthening the manuscript. In this revision, we have addressed all concerns raised. Additionally, we refined the text of some paragraphs to enhance clarity and readability without altering our core findings, and highlighting every modification. Finally, we moved all supplementary tables to a separate Excel file to further improve readability of the manuscript.

Reviewer #1 (Remarks to the Author):

I appreciate the authors' dedication to this revision. The authors addressed my comments well.

I have one more comment. In Discussion, the authors state that "Moreover, for the first time, our approach is the only that can analyse datasets with over 10 million cells and thousands of genes efficiently."

I find the recent pseudobulk-based DE tool "dreamlet" attractive when analyzing millions of cells, as it takes only a few minutes for 1M cells. Thus, the above sentence could be overstatement. Although superior accuracy of devil over pseudobulk methods are shown well in Fig 2, such advantage could be rather reduced when analyzing millions of cells. Is there computational bottleneck to apply pseudobulk methods to 10M cells? Pros and cons between pseudobulk method like dreamlet and devil need to be stated to clearly guide users when and why to use this tool.

We thank the reviewer for raising this point and agree that the original phrasing was an overstatement. We have revised the sentence to temper the claim and explicitly acknowledge the trade-offs between cell-level and pseudobulk approaches. Specifically, we now note that while pseudobulk tools are highly efficient (since their computational costs scales with the number of donors rather than cells), this design choice comes at the cost of discarding within-patient cell-to-cell variability. Moreover, by aggregating cells into pseudo-samples, statistical power in these models is driven by the number of patients, not the number of cells, meaning that adding more cells does not inherently improve inference.

Other minor comments:

The numbers of cells used in each study need to be stated in legends of Suppl Fig 7~10.

We have added the number of cells used in each simulated dataset to the captions of Supplementary Figures 7–10.

Line 218 needs revision

We have revised line 218 accordingly.

What happens to MAST curves in Fig. 2D?

We thank the reviewer for detecting this. The MAST curve in Figure 2D was incorrectly plotted due to a computational error; this has now been corrected.

Legend of Fig 2CD needs revision

We revised the legend of Fig 2CD to improve clarity.

Ref 31. Preprint

Thank you, reference 31 now explicitly states that we are referring to a preprint.

Reviewer #2 (Remarks to the Author):

I appreciate the authors great improvements of the manuscript and have only a few remaining comments:
Figure 2: Figure 2 C and D - benchmarking: the different models are not so easily distinguished or out of frame, a comprehensive overview (maybe in the supplement) might help to show the data more fully. Additionally, while the extended figure clearly shows the speed-up of devil compared to NEBULA, I think it is important to highlight this in Figure 2A, as in other metrics (MCC and TPR QQ-Plots), NEBULA shows the highest similarity to devil. Thus, to strengthen the advantages of devil, I would more clearly highlight the comparison and the benefits of devil.

Thank you for this suggestion. We have added specific quantitative metrics to the figures, namely lambda inflation factors and AUC values, which help highlight differences between tools that would otherwise be difficult to distinguish visually. We have also added a supplementary figure (SF.10) reporting these metrics comprehensively across datasets, models, and number of input samples, further stratifying results by method class (pseudobulk-based, single-cell-based, and single-cell with patient integration). Additionally, following your comment on the importance of the devil–NEBULA comparison, we have added a dedicated scaling benchmark against NEBULA directly in Figure 2A, more clearly showing devil's computational advantages.

P.9 l.244 possible typo: 5 markers

We have revised the sentence to clarify that we report two distinct metrics: peak accuracy and peak average accuracy across p-value thresholds, meaning that different tools might have peak accuracies for different numbers of markers.

Extended Figure 3: Please adjust the heatmap to a colour scheme that is not red-green (e.g. use blue instead of green)

The heatmap colour scheme has been updated from red-green to red-blue, improving readability and accessibility for colour-blind readers.

P.13 l.357 typo/ missing adjustment: 10 million cells

Thank you for noticing it. The type has been corrected.

Lastly to make this upgraded method more accessible to potential users, I think it should be noted in the manuscript, that this method is available as an R package. Additionally computational requirements and R versions should be listed as is common when publishing a new package.

We have added richer code availability statements, reporting the versions of the packages used.

Reviewer #3 (Remarks to the Author):

The authors thoroughly addressed all the comments in my previous review and I am satisfied with the current version of the manuscript.

I only have the following minor points that are needed to improve the clarity of the presentation:

1. Please specify in the text the meaning of the multiplication operator in equation 1, as it is not a standard notation in bioinformatics.

We have clarified the notation in the main text, specifying that the operator refers to the Hadamard (element-wise) product.

2. In responding to a comment by another reviewer, the authors have implemented a new approach for the estimation of the dispersion parameter based on the method of

moments. If I understand correctly all the results presented in this version of the manuscript are obtained using this approach. It would be useful for a reader to show in a supplementary figure or table that the two estimators achieve qualitatively the same results in practice.

We have added a new Supplementary Figure (SF.11) comparing the two dispersion estimators across simulated scenarios. As shown, both approaches yield qualitatively and quantitatively equivalent results in terms of accuracy, while the method-of-moments estimator provides a substantial speed improvement. A brief reference to this figure has been added to the main text.

3. In Section 4.9 the authors state that "the wall-clock time scale with the number of GPUs, reaching a maximum speedup of 5.2 using 8 GPUs." The speedup of 5.2 is compared to what? A single GPU? Please clarify.

We have clarified the text to state explicitly that the reported speedup of 5.2× is relative to a single-GPU baseline.

Reviewer #3 (Remarks on code availability):

While it is straightforward to install the package without GPU support, unsurprisingly, I struggeled installing with CUDA support.

The authors provide good guidelines in two dedicated vignettes, but I suggest to add this content (or a link to the vignettes) in the README, as most users would expect to find detailed installation instructions there.

Thank you for raising this lack of clarity. We now refer to the vignettes directly in the README.

Reviewer #4 (Remarks to the Author):

I co-reviewed this manuscript with one of the reviewers who provided the listed reports. This is part of the Nature Communications initiative to facilitate training in peer review and to provide appropriate recognition for Early Career Researchers who co-review manuscripts.

RESPONSE TO REVIEWERS' COMMENTS

We thank the reviewers for their positive feedback. In this last revision we refined the text of some paragraphs to enhance clarity, readability and to comply with editorial requirements without altering any findings. Similarly, to comply with the required format, we turned two Extended Data Figures to Main Figures.

Reviewer #1 (Remarks to the Author):

The authors addressed all my comments.

Reviewer #2 (Remarks to the Author):

The authors addressed the comments well and I am satisfied with the current version of the manuscript.

Reviewer #3 (Remarks to the Author):

The authors satisfactorily addressed my remaining comments. I congratulate the authors on a nice paper.

Reviewer #4 (Remarks to the Author):

I co-reviewed this manuscript with one of the reviewers who provided the listed reports. This is part of the Nature Communications initiative to facilitate training in peer review and to provide appropriate recognition for Early Career Researchers who co-review manuscripts.
