# Supplementary material for: Scalable, fast and accurate differential gene expression testing from millions of cells of multiple patients
Source: Nat Commun. 2026 Jun 19;17:7734. doi: 10.1038/s41467-026-74451-9 (PMC13434233; doi:10.1038/s41467-026-74451-9)
Supplement: Supplementary file 3 [file 41467_2026_74451_MOESM3_ESM.pdf]

nature portfolio

Corresponding author(s): Giulio Caravagna

Last updated by author(s): May 11, 2026

Reporting Summary

Nature Portfolio wishes to improve the reproducibility of the work that we publish. This form provides structure for consistency and transparency in reporting. For further information on Nature Portfolio policies, see our Editorial Policies and the Editorial Policy Checklist.

Statistics

For all statistical analyses, confirm that the following items are present in the figure legend, table legend, main text, or Methods section.

n/a	Confirmed	
☐	☒	The exact sample size (*n*) for each experimental group/condition, given as a discrete number and unit of measurement
☐	☒	A statement on whether measurements were taken from distinct samples or whether the same sample was measured repeatedly
☐	☒	The statistical test(s) used AND whether they are one- or two-sided<br>*Only common tests should be described solely by name; describe more complex techniques in the Methods section.*
☐	☒	A description of all covariates tested
☐	☒	A description of any assumptions or corrections, such as tests of normality and adjustment for multiple comparisons
☐	☒	A full description of the statistical parameters including central tendency (e.g. means) or other basic estimates (e.g. regression coefficient) AND variation (e.g. standard deviation) or associated estimates of uncertainty (e.g. confidence intervals)
☐	☒	For null hypothesis testing, the test statistic (e.g. *F*, *t*, *r*) with confidence intervals, effect sizes, degrees of freedom and *P* value noted<br>*Give P values as exact values whenever suitable.*
☒	☐	For Bayesian analysis, information on the choice of priors and Markov chain Monte Carlo settings
☒	☐	For hierarchical and complex designs, identification of the appropriate level for tests and full reporting of outcomes
☒	☐	Estimates of effect sizes (e.g. Cohen's *d*, Pearson's *r*), indicating how they were calculated

Our web collection on statistics for biologists contains articles on many of the points above.

Software and code

Policy information about availability of computer code

Data collection	All the relevant data analysed in this work was obtained from public data resources with no use of software.
Data analysis	The DEVIL framework is implemented in R and is available as an open-source R/C++ package at https://github.com/caravagnalab/devil and the frozen verison used for the analysis is available at https://zenodo.org/records/20068507 . The code to replicate the analysis of this paper, along with data accession links, are available at https://zenodo.org/records/20068224. Differential expression testing was carried out using edgeR v4.2.2, limma v3.60.6, glmGamPoi v1.16.0, NEBULA v1.5.6, and MAST v1.30.0. Single-cell data processing and clustering were performed with Seurat v5.4.0 and SingleCellExperiment v1.26.0. Data manipulation and visualisation relied on the tidyverse v2.0.0, including ggplot2 v4.0.2, dplyr v1.2.0, tidyr v1.3.2, and purrr v1.2.1, together with patchwork v1.3.2 and ggh4x v0.3.1 for figure assembly.

For manuscripts utilizing custom algorithms or software that are central to the research but not yet described in published literature, software must be made available to editors and reviewers. We strongly encourage code deposition in a community repository (e.g. GitHub). See the Nature Portfolio guidelines for submitting code & software for further information.

Data

Policy information about availability of data

All manuscripts must include a data availability statement. This statement should provide the following information, where applicable:

- Accession codes, unique identifiers, or web links for publicly available datasets
- A description of any restrictions on data availability
- For clinical datasets or third party data, please ensure that the statement adheres to our policy

No new data were generated during the study. The data that support the findings of this study are all publicly available. The accession codes for all the analyzed datasets are available using the README file available at https://zenodo.org/records/20068224

Research involving human participants, their data, or biological material

Policy information about studies with human participants or human data. See also policy information about sex, gender (identity/presentation), and sexual orientation and race, ethnicity and racism.

Reporting on sex and gender	This study analysed previously published single-cell RNA-sequencing datasets from human donors. Information on biological sex was available only for a subset of datasets and was not consistently reported across sources. Sex and gender were not variables of interest in this study, and no analyses were stratified by sex or gender. Gender identity information was not available.
Reporting on race, ethnicity, or other socially relevant groupings	This study analysed previously published single-cell RNA-sequencing datasets. Information on race, ethnicity, or other socially relevant groupings was not available or not consistently reported in the original data sources and was therefore not considered in the analyses.
Population characteristics	This study analysed previously published single-cell RNA-sequencing datasets from human donors. Age information was available and explicitly modelled in one dataset (the skeletal muscle ageing analysis), where it was included as a covariate in the statistical design; no additional population stratification was performed.
Recruitment	No participant recruitment was performed in this study. All analyses were conducted on previously published datasets, and recruitment procedures, inclusion criteria, and consent processes were defined by the original studies.
Ethics oversight	All analyses were conducted on previously published datasets and protocol approvals were defined by the original studies.

Note that full information on the approval of the study protocol must also be provided in the manuscript.

Field-specific reporting

Please select the one below that is the best fit for your research. If you are not sure, read the appropriate sections before making your selection.

☒ Life sciences ☐ Behavioural & social sciences ☐ Ecological, evolutionary & environmental sciences

For a reference copy of the document with all sections, see nature.com/documents/nr-reporting-summary-flat.pdf

Life sciences study design

All studies must disclose on these points even when the disclosure is negative.

Sample size	No prospective sample size calculation was performed for this study. Sample sizes were determined by the availability of previously published single-cell RNA-sequencing datasets and are reported in the corresponding original studies.
Data exclusions	Data were excluded only through standard pre-processing filters based on gene- and cell-level quality metrics, as described in the Methods section.
Replication	All analyses were performed across multiple independent, previously published datasets. Methodological results were evaluated through repeated simulations and benchmarking experiments as described in the Methods.
Randomization	No randomization was performed in this study, as analyses were conducted on previously published datasets.
Blinding	Blinding was not applicable to this study, as all analyses were computational and performed on pre-existing datasets with known experimental annotations.

Reporting for specific materials, systems and methods

We require information from authors about some types of materials, experimental systems and methods used in many studies. Here, indicate whether each material, system or method listed is relevant to your study. If you are not sure if a list item applies to your research, read the appropriate section before selecting a response.

Materials & experimental systems

n/a	Involved in the study
☒	☐ Antibodies
☒	☐ Eukaryotic cell lines
☒	☐ Palaeontology and archaeology
☒	☐ Animals and other organisms
☒	☐ Clinical data
☒	☐ Dual use research of concern
☒	☐ Plants

Methods

n/a	Involved in the study
☒	☐ ChIP-seq
☒	☐ Flow cytometry
☒	☐ MRI-based neuroimaging

Plants

Seed stocks	*Report on the source of all seed stocks or other plant material used. If applicable, state the seed stock centre and catalogue number. If plant specimens were collected from the field, describe the collection location, date and sampling procedures.*
Novel plant genotypes	*Describe the methods by which all novel plant genotypes were produced. This includes those generated by transgenic approaches, gene editing, chemical/radiation-based mutagenesis and hybridization. For transgenic lines, describe the transformation method, the number of independent lines analyzed and the generation upon which experiments were performed. For gene-edited lines, describe the editor used, the endogenous sequence targeted for editing, the targeting guide RNA sequence (if applicable) and how the editor was applied.*
Authentication	*Describe any authentication procedures for each seed stock used or novel genotype generated. Describe any experiments used to assess the effect of a mutation and, where applicable, how potential secondary effects (e.g. second site T-DNA insertions, mosiacism, off-target gene editing) were examined.*
