# Supplementary material for: Scalable, fast and accurate differential gene expression testing from millions of cells of multiple patients
Source: Nat Commun. 2026 Jun 19;17:7734. doi: 10.1038/s41467-026-74451-9 (PMC13434233; doi:10.1038/s41467-026-74451-9)
Supplement: Supplementary file 4 [file 41467_2026_74451_MOESM4_ESM.pdf]

Description of Additional Supplementary Files

Supplementary Data 1: **Representative single-cell differential expression frameworks.** Comparison of representative single-cell differential expression (scDE) frameworks. Variables are number of cells N; number of genes G; number of patients P; number of covariates F, and embedding dimension D.

Supplementary Data 2: **Summary of public datasets.** Summary of the datasets analyzed in our work. Each entry reports the original publication and the accession code for the download.

Supplementary Data 3: **Scaling results over Chiou et al. dataset.** Runtime and total memory allocation for DE analysis of the dataset presented by Chiou et al.

Supplementary Data 4: **Scaling results over Baron et al. dataset.** Runtime and total memory allocation for DE analysis of the dataset presented by Baron et al.

Supplementary Data 5: **Differential expression analysis methods benchmarked.** Summary of differential expression (DE) models benchmarked in this study. Methods are grouped by data granularity (pseudobulk vs. cell-level) and by their statistical formulation. NB stands for Negative Binomial, CPM for count per millions and MLE for Maximum-Likelihood-Estimator.

Supplementary Data 6: **Performance over cell-wise simulations.** For each dataset and patient size, the median Matthews correlation coefficient (MCC) is reported along with 05-th and 95-th quantile.

Supplementary Data 7: **Performance over patient-wise simulations.** For each dataset and patient size, the median Matthews correlation coefficient (MCC) is reported along with 5th and 95th quantile.

Supplementary Data 8: **Computational cost for cell- and patient-wise simulations.** Computational time performance in seconds for DE analysis. For each dataset and number of cells, the median time is reported along with standard deviation.

Supplementary Data 9: **Computational fold change for cell- and patient-wise simulations.** Computational time ratio with respect to devil (MOM-NB) for DE analysis. For each dataset and number of patients, the median time is reported along with 5th and 95th quantile.

Supplementary Data 10: **Hardware specification.** Hardware specifications and software environment of the computational resources used in the experiments (ORFEO cluster).
